# Tracking the Breakdown of Quantum Confinement during Structural Degradation of FAPbI_3_

**DOI:** 10.1021/acs.jpclett.6c01253

**Published:** 2026-05-27

**Authors:** Gurpreet Kaur, Sarah J. Scripps, Joshua R. S. Lilly, Nakita K. Noel, Michael B. Johnston, Laura M. Herz

**Affiliations:** Department of Physics, 6396 University of Oxford, Clarendon Laboratory, Oxford OX1 3PU, United Kingdom

## Abstract

Bulk formamidinium lead triiodide (FAPbI_3_) films host spontaneously formed quantum-confined (QC) domains, but their structural origin remains unclear. Using controlled material degradation in humid air as a dynamic lattice perturbation, we track the evolution of QC features in thin-film absorption of FAPbI_3_. With aging, above-bandgap QC features redshift and diminish, indicating weakened electronic confinement. Concurrently, X-ray diffraction reveals that breakdown of α-phase connectivity coincides with the loss of short-range higher-order hexagonal (nH, *n* > 2) polytypes as the material converts to the 2H δ-phase. Such polytypic nanodomains may generate peaked absorption features by forming higher-energy barriers confining charge carriers within α-FAPbI_3_ or by introducing distinct electronic states associated with mixed octahedral connectivity. Progressive degradation dismantles this framework, causing the disappearance of the QC features. Our results identify the structural motifs underpinning QC effects and propose that controlling higher-order (*n* > 2) hexagonal polytypes offers a route to tuning quantum confinement in FAPbI_3_ films.

## Graphical abstract

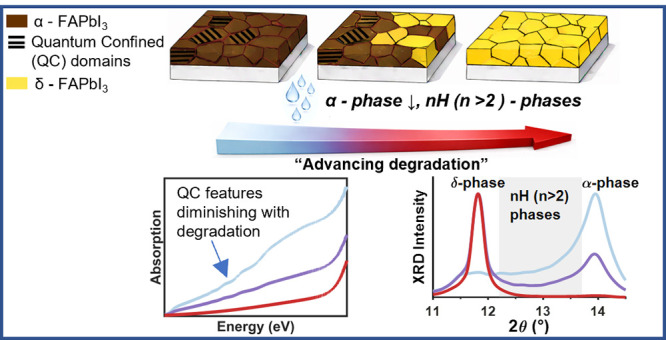

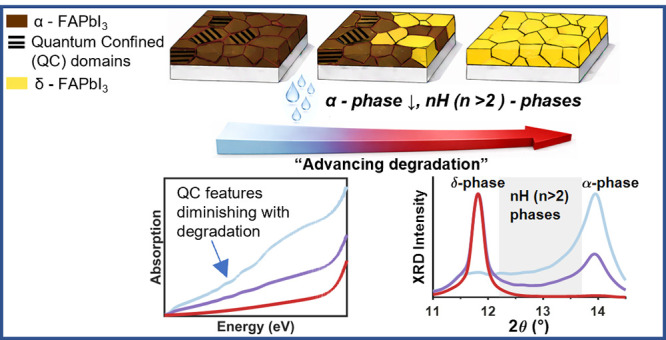

Formamidinium lead triiodide (FAPbI_3_) is widely regarded as a benchmark metal halide perovskite absorber for photovoltaic applications. In its photoactive α-phase, it combines a near-optimal bandgap of ∼ 1.48 eV with a high optical absorption coefficient (≥10^4^ - 10^5^ cm^–1^ across the visible region of the spectrum), excellent charge-carrier mobilities, long diffusion lengths, and an electronic structure inherently tolerant to defects.^1–10^ While its methylammonium based counterpart (MAPbI_3_) shares many of these favorable characteristics, α-FAPbI_3_ exhibits significantly enhanced thermal stability, which directly translates into improved long-term device reliability under operational conditions.^6^ This unique combination of properties underpins its outstanding optoelectronic quality and explains why α-FAPbI_3_ has consistently enabled record-setting power conversion efficiencies in photovoltaic devices.^11–13^ Yet, these appealing properties coexist with inherent structural fragility. The desired α-phase is only metastable under ambient conditions and exhibits a thermodynamic preference toward the wide-bandgap δ-phase (hexagonal 2H polytype, indirect band gap ∼ 2.5 eV), creating a major challenge for practical stability.^14–17^

In addition to this well-known phase instability, a distinct feature of FAPbI_3_ that has recently garnered attention is its propensity to form self-assembled nanostructures, even within nominally bulk films,^18–22^ which manifest themselves as distinct peak features in the absorption spectrum at energies above the bandgap of FAPbI_3_. The energetic spacing between consecutive peaks shows a quadratic dependence, hallmark signatures of transitions between quantum-confinement (QC) levels in a potential well or periodic superlattice, with length scales of 10–20 nm.^18^ Temperature-dependent measurements have further revealed that the associated energetic shifts scale inversely with the square of the reduced lattice parameters for the FAPbI_3_ perovskite α-phase, pointing to the α-phase framework as the likely origin.^18^

Despite these intriguing initial findings, the central question of which structural entities result in these peaked absorption features remains unresolved. It has been hypothesized that quantum well barriers originate from local strain fields, polytypic or other nanoscale heterogeneities within the α-phase network, yet their precise structural identity remains experimentally unresolved.^18,20–22^ Meta-analyses comparing photovoltaic devices with and without QC signatures indicate that these domains correlate with reduced device efficiency.^21^ This reduction can be readily rationalized, as spatially localized nanodomains impose discontinuous potentials that impede charge transport within the perovskite bulk.^21^ Thus, understanding these confined structures is essential for developing effective mitigation strategies and holds clear technological significance.

In this context, the situation is further complicated by the propensity of FAPbI_3_ to exhibit pronounced polytypism. During the δ ⇔ α transformation, a series of mixed hexagonal stacking sequences (e.g., 4H, 6H, and 8H) can be accessed, in which face- and corner-sharing PbI_6_ octahedra alternate in polytype-specific ratios, in contrast to the purely face-sharing 2H (δ-phase) and purely corner-sharing 3C (α-phase) limits.^23–27^ Notably, crystallographic studies have shown that such mixed hexagonal stacking sequences can also occur as local intergrowths or stacking faults within material otherwise indexed as α-FAPbI_3_. Since an increased proportion of face-sharing octahedra is systematically associated with wider band gaps,^27,28^ the presence of mixed stacking sequences within the α-phase may give rise to spatial variations in the local electronic structure. Such stacking heterogeneity therefore provides a plausible route for generating higher-bandgap regions within the perovskite matrix.

In this study, we exploit such lattice perturbation during gradual α- to δ- phase transformation of FAPbI_3_ to explore which type of structural motifs may give rise to the observed QC absorption features. We show that such slow degradation under a carefully chosen humid environment progressively generates δ domains while gradually altering polytypic sequences, stacking coherence, and ultimately the local bandgap contrast affecting QC. By simultaneously tracking above-bandgap QC signatures in absorption, while monitoring crystallographic evolution revealed by X-ray diffraction, degradation serves as a direct diagnostic of the confinement landscape. The results point to two plausible interpretations: (1) the QC wells are located within α-FAPbI_3_ domains, with the surrounding barriers arising from higher-order polytypes (nH, *n* > 2); or (2) the polytypes themselves host discrete electronic states that reflect a periodic superlattice nature. Aging progressively disrupts this QC architecture, leading to a weakening of the QC signatures, while the monotonic energy shifts of the confined states with progressing degradation are consistent with a gradually diminishing confinement strength. Overall, this coupled optical - structural approach links the evolution of confined states to lattice reorganization and identifies the motifs that define QC effects in FAPbI_3_.

We employed solution processed FAPbI_3_ films, with the full preparation procedure described in the Supporting Information. We note that peaked absorption features arising from QC have been observed across FAPbI_3_ films processed in a variety of ways, including vapor-phase evaporation,^18^ solution-processing and aerosol-assisted recrystallization.^20,22^ To follow their concurrent optical and structural evolution during degradation, steady-state absorption spectra and XRD patterns were recorded under controlled humidity exposure (85% relative humidity (RH) at room temperature). Elevated humidity was used to accelerate the α- to δ-phase transformation, allowing all measurements to be completed within experimentally practical time scales of 50 h.

Monitoring the absorption spectra over time, we observe a progressive loss in overall absorption strength as degradation advances, with the α-FAPbI_3_ (3C phase) band edge gradually fading (Figure 1A). Interestingly, this decline is accompanied by simultaneous loss of the characteristic high-energy QC peak features. To quantify this evolution, a spline baseline was subtracted from each absorption coefficient spectrum (as illustrated in the inset of Figure 1A), isolating the QC contribution from the continuum and enabling a direct, background-independent comparison of strength of the peak features, consistent with our methodology reported earlier.^18,20^ As Figure 1B shows, the isolated QC features diminish in amplitude in a gradual and systematic manner, vanishing entirely after 47 h of high-humidity exposure, at which point the FAPbI_3_ α-phase has fully converted to the thermodynamically favored δ- (2H) phase.

**Figure 1:**
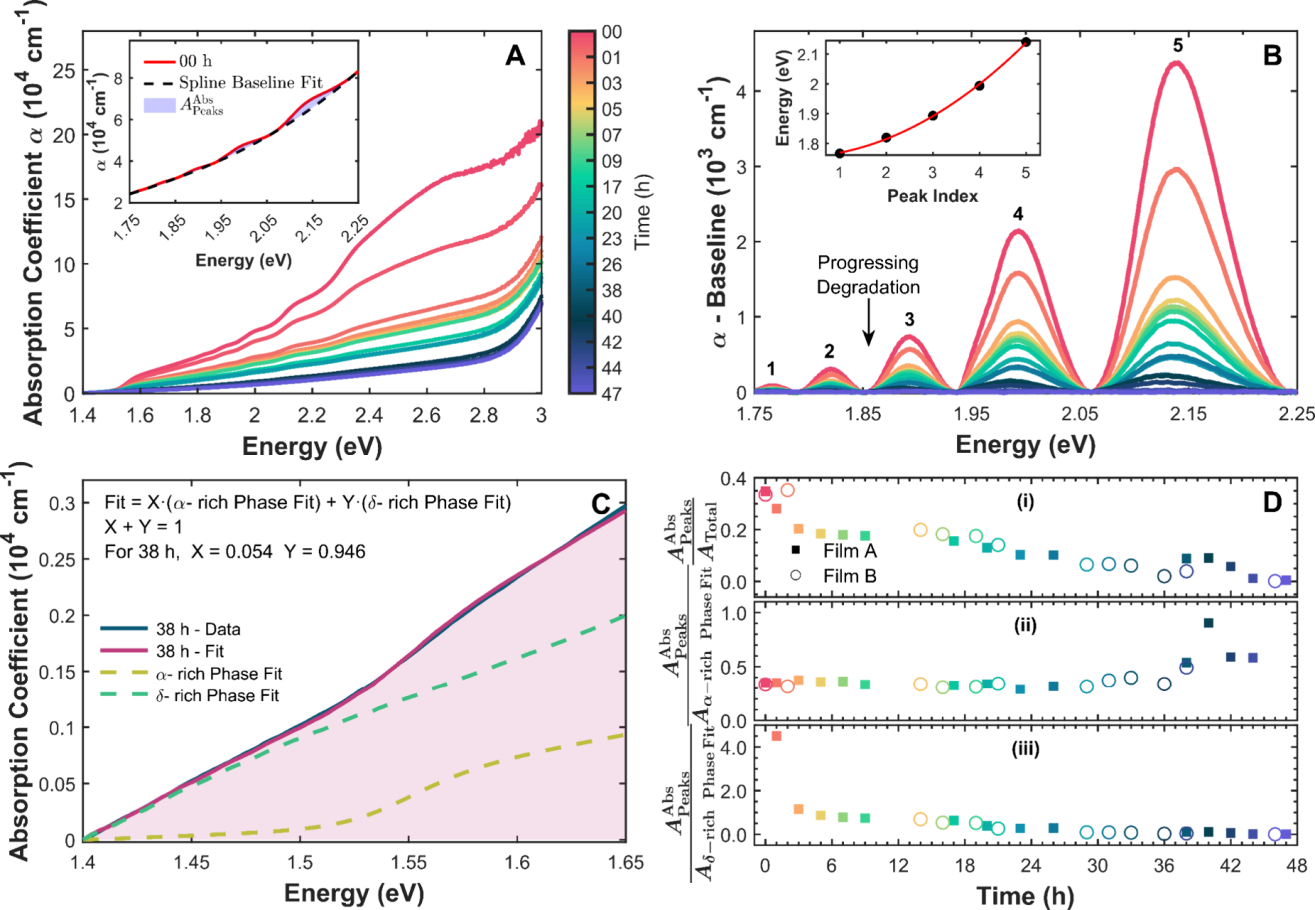
(A) Absorption coefficient spectra of a FAPbI_3_ film during exposure to 85% RH at room temperature. Inset: representative spline baseline used to isolate the oscillatory QC features. (B) Baseline-subtracted QC features as a function of aging time. Inset: quadratic scaling of confined-state peak energies with peak index for the initial 0 h spectrum. (C) Representative linear decomposition of the 38h spectrum within the α-FAPbI_3_ band-edge window, 1.4–1.65 eV, using fixed optical endmembers associated with the initial α-rich state and the final δ-rich degraded state. (D) Integrated QC peak areas, (*A*_*Peaks*_^*Abs*^), normalized to the integrated areas of the α-associated optical contribution (A_α‑rich Phase Fit_), the δ-rich degraded-state optical contribution, (A_δ‑rich Phase Fit_) and the total absorption (A_Total_), evaluated within the same decomposition window. Filled squares correspond to FAPbI_3_ Film A and open circles to Film B.

To quantify the relative phase contributions during the transformation process, each time-resolved absorption spectrum was expressed as a weighted linear combination of two reference spectra representing the α-rich phase (initial state, 0 h) and the degraded 2H δ-phase rich state (final state, 47 h). For all intermediate spectra, we performed a constrained linear decomposition into these α- and δ-phase endmembers (see Figures S1, S2, and S3) over the region near the band edge (1.4 – 1.65 eV). This narrow energy window was chosen to avoid absorption contributions from higher-order hexagonal polytypes (nH, *n* > 2) which exhibit reduced Pb–I orbital overlap and, consequently, a larger bandgap compared to the corner-sharing α (3C) phase.^29,30^ As discussed below, such higher order polytypes are initially present in significant amounts in the films studied here but gradually diminish during degradation, underscoring the need of this caution. A representative fit for the data set recorded after 38 h in humid air (chosen arbitrarily for illustration) is shown in Figure 1C. At this stage, the fit indicates that the α-associated optical contribution has decreased to ∼ 5.4%, while the δ-rich degraded-state contribution accounts for the remaining ∼ 94.6% within the selected fitting window. Equivalent linear decomposition analyses for all time points (Figure S1), together with the time evolution of the extracted optical contributions (Figure S3, Table S1), are provided in the Supporting Information.

We now use these quantitative measures of the relative presence of either α-rich or δ-rich optical contributions to explore whether either quantity correlates with the magnitude of the peaked features attributed to quantum confinement. As a measure of the QC signatures, we use the spectrally integrated area under the isolated high energy features, and compare this to the integrals for the α-rich and 2H δ-rich contributions to the overall absorption spectrum. Figure 1D shows the spectral contributions from the QC absorption peaks (*A*_*Peaks*_^*Abs*^) divided by either the integrated area for the original α-rich fit (A_α‑rich Phase Fit_), or the 2H δ-rich degraded-state fit (A_δ – rich Phase Fit_) or the total (A_Total_). The individual spectral areas are provided in Figure S3. Interestingly, these ratios highlight a clear positive corelation between the presence of the original perovskite α-rich optical contribution and the QC absorption peaks: Figure 1D shows that the ratio *A*_*Peaks*_^*Abs*^/A_α‑rich Phase Fit_ remains largely unchanged throughout the period until the film has completely transformed into the δ-rich degraded state. In contrast, we see that while the quantity *A*_*Peaks*_^*Abs*^/*A*_Total_ declines over time and *A*_*Peaks*_^*Abs*^/ A_δ‑Phase Fit_ decreases, suggesting that the presence of 2H δ-phase is not a positive requirement for the formation of these QC features. Instead, our analysis highlights an association between the high-energy QC features and the presence of α-phase (3C). We note that this finding is repeatable between individual films of the fabricated set; Figure 1D shows metrics obtained for FAPbI_3_ “Film A” in full squares and for a second film (“Film B”) in open circles, which show good agreement.

We emphasize that this linear decomposition is used as an empirical optical tracking method rather than as a quantitative crystallographic phase-fraction analysis. Accordingly, the α- and δ-phase contributions should be understood as optical endmember contributions associated with the α-FAPbI_3_ band-edge response and the δ-rich degraded film state, respectively. In the selected 1.4–1.65 eV fitting window, the δ-rich contribution should not be interpreted as intrinsic interband absorption of ideal 2H δ-FAPbI_3_, whose fundamental absorption onset lies at higher energy. Instead, it represents the optical response of the degraded δ-rich film state in this spectral region, including possible contributions from sub-bandgap tail absorption, scattering losses, disorder-induced absorption, and residual structural heterogeneity. To test the robustness of this interpretation, we repeated the decomposition over an extended 1.4–3.0 eV range, where optical features from the δ-rich degraded state contribute more strongly. This extended-range analysis, shown in Figures S4–S6, gives the same qualitative evolution, confirming that the decrease of the α-associated optical contribution, the increase of the δ-rich degraded-state contribution, and the disappearance of the QC peaks are retained when the fitting range is expanded beyond the near-band-edge window.

To explore whether the presence of QC features arises directly from the α-phase of FAPbI_3_ or might be correlated with structural polymorphs that coexist with the α-phase, we also recorded XRD patterns (Figure 2A) at successive stages of degradation. The film initially shows no detectable δ-phase, but as degradation onsets, the α-phase (3C) reflections (gray dashed lines) systematically diminish while the δ-phase (2H) reflections (black dashed lines) increase. At later stages, additional peaks appear corresponding to hydrate phases (black solid lines) formed under prolonged high-humidity exposure, consistent with previous reports on FAPbI_3_ aged under humid conditions.^31^ The temporal evolution of all major reflections is provided in the Supporting Information (Figure S8). Closer inspection of the region between the first-order δ- and α-phase reflections reveals a broad diffuse background in the as-prepared films, centered around 2θ = 12.2–13.6° (Figure 2B). To confirm that this broad feature is not an instrumental artifact or background contribution from the substrate, we compared the XRD pattern of a bare quartz substrate with that of the as-prepared FAPbI_3_ film (Figure S7). The broad diffuse feature observed in the FAPbI_3_ film is not present in the bare substrate measurement, supporting its assignment to a diffuse scattering contribution originating from the film. Similar diffuse profiles have been documented in polytypic systems such as β - SiC, where correlated stacking faults and mixed-layer sequences produce enhanced background intensity and apparent peak broadening without distinct phase formation.^32,33^ By analogy, the diffuse background in FAPbI_3_ likely originates from short-range stacking deviations, such as thin 4*H*/6H/8H like lamellae or faulted fragments embedded within the α-framework. This interpretation is supported by the fact that reflections from such motifs fall within the 2θ range expected for these higher-order polytypes^30^ (Figure S9), even though distinct peaks are absent.

**Figure 2:**
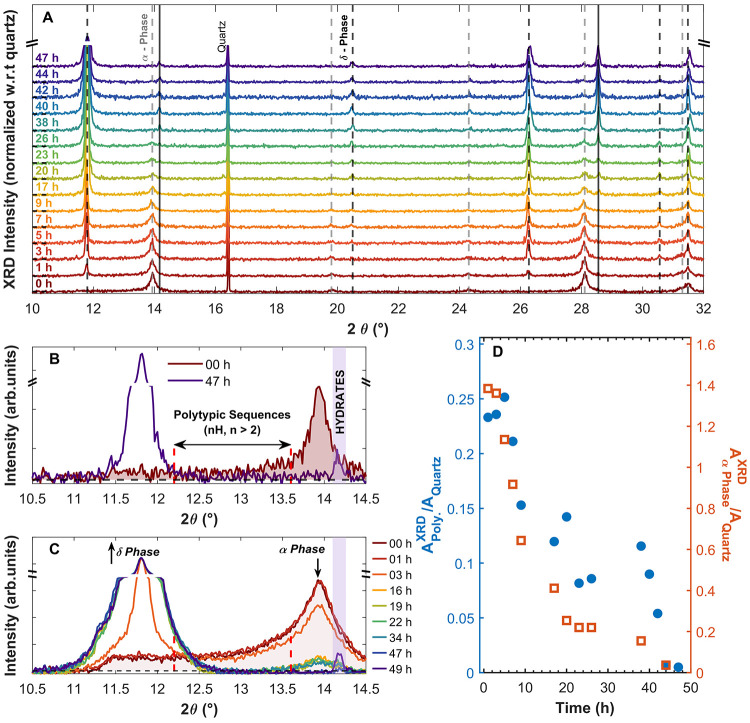
(A) XRD patterns recorded on PANalytical X’Pert PRO during humidity-induced α → δ transformation. Vertical gray dashed lines denote α-phase reflections, black dashed lines indicate δ-phase reflections, and black solid lines correspond to hydrate peaks. (B) Magnified region from (A) highlighting the diffuse background between 2θ = 12.2–13.6°, bounded by red dashed lines; horizontal dashed lines indicate the baseline reference, and the light violet-shaded area marks the hydrate-related contribution. (C) Corresponding measurements acquired on a Rigaku SmartLab diffractometer (different film). (D) Integrated areas under the α-phase reflections and the diffuse background plotted versus aging time, derived from (A).

With increasing humidity exposure, this diffuse component decreases alongside the α-phase reflections, indicating concurrent loss of both long-range α-phase order and short-range higher-order polytypic disorder. When we examined such effects on two films fabricated under identical conditions but two different diffractometers, a PANalytical X’Pert PRO (Figure 2B) and a Rigaku SmartLab diffractometer (Figure 2C), both show the same qualitative behavior. We note that the Rigaku SmartLab’s wide-acceptance stationary detector is particularly sensitive to weak off-axis scattering, allowing such diffuse signals associated with local stacking irregularities to be detected with particular ease (see Figure 2C). However, because this geometry yields orientation dependent patterns (see SI) we base our quantitative analysis on data recorded by the PANalytical X’Pert PRO system, whose continuous sample spinning provides an orientation averaged and reproducible diffraction response. Integrated intensities (Figure 2D), normalized to the quartz reference, show that both the α-phase and such diffuse contributions diminish together, indicating that polytypic 4*H*/6H/8H motifs that initially delineate α domains also vanish during structural reorganization.

Taken together, the XRD data indicate that as-prepared FAPbI_3_ films exhibiting QC signatures contain short-range polytypic modulations or thin hexagonal lamellae embedded within the α-phase lattice. One way these higher-order polytypes may cause QC is by serving as barriers, confining charge carriers in quantum wells formed by the 3C perovskite α-phase. Because these higher-order hexagonal polytypes possess significantly wider bandgaps than the α-phase,^30^ the resulting band offset enables polytypic lamellae to localize charge carriers in adjacent α-phase regions, giving rise to QC features. Alternatively, some of the higher-order polytype motifs may exhibit an intrinsic structural periodicity arising from the stacking of octahedral networks with differing connectivity, producing periodically as well as spatially modulated bandgaps. In this case, the above-gap peaks attributed to QC may arise within the polytypes themselves through a self-modulated potential landscape. Upon degradation, these irregularities disappear as the α-framework reorganizes, progressively eliminating higher-order (4H/6H/8H) polytypes. In contrast, the growth of the 2H δ-phase under humid air clearly weakens QC features, showing that this particular polytype is not the cause of the QC signatures. The coupled optical and structural evolution therefore supports a confinement picture in which α-phase domains are bounded by neighboring higher-order polytypic regions that act as barriers, or alternatively where the peaked features arise from the periodic band structure within these regions themselves.

We further probe how the gradual degradation of FAPbI_3_ in humid air affects the electronic signature of the peaked absorption features attributed to QC. Close examination of the temporal evolution, as shown in Figure 3A for two representative peaks, reveals that peaks shift to lower energy with time, with the higher-index peak exhibiting larger changes. This behavior is quantified in Figure 3B which tracks the energy of the peak maxima over time, relative to their initial (0 h) value (see SI for evaluation details and absolute shifts). This figure clearly reveals that the highest-index peaks at the largest energies are the most sensitive to structural evolution. These transitions would be the least confined inside a quantum well or a periodic superlattice, and therefore any changes to electronic barrier states would indeed be expected to have the greatest effect.

**Figure 3:**
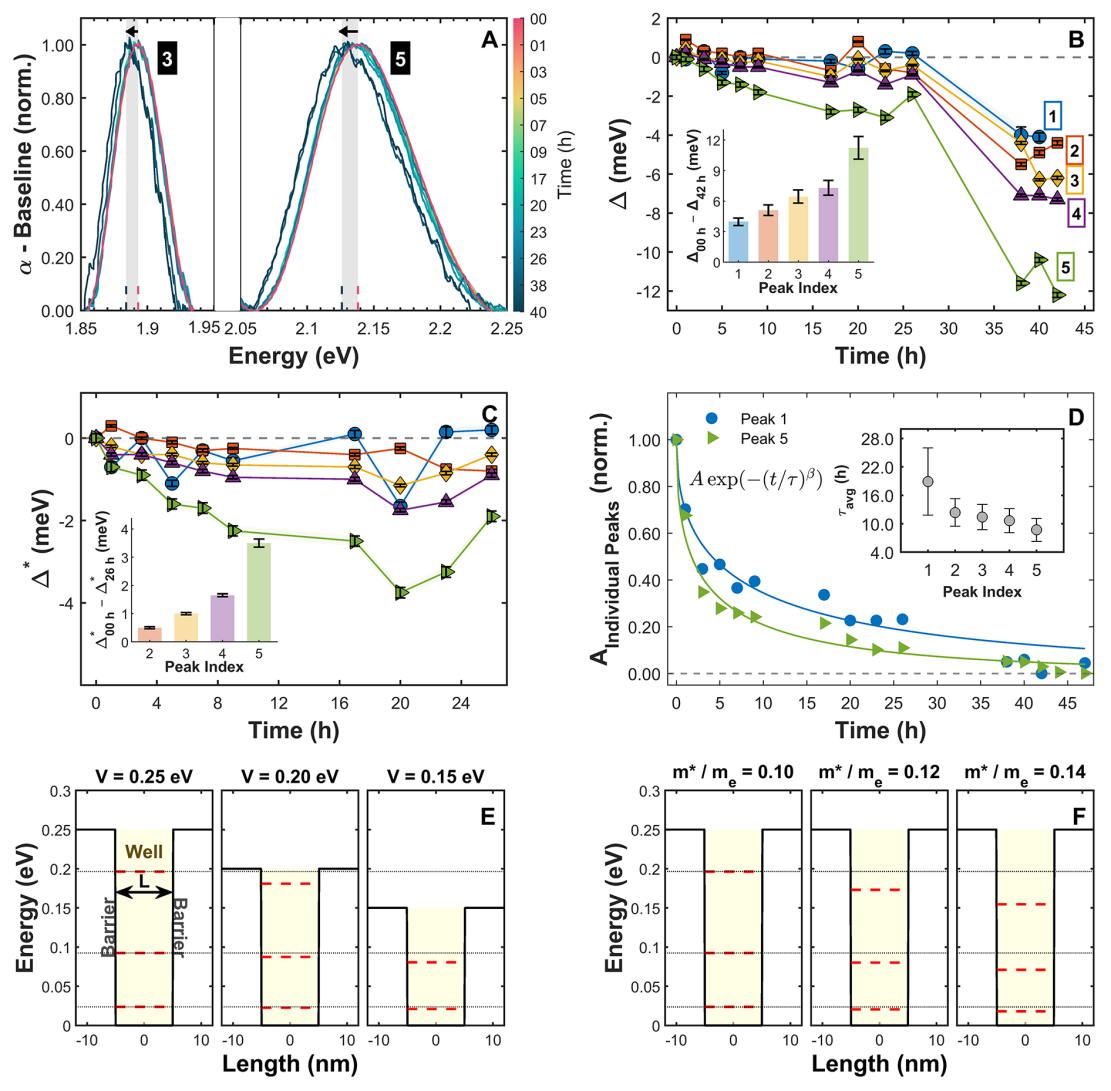
(A) Background subtracted features highlighting Peak 3 and 5. (B) Peak maxima shifts, denoted by Δ, as referenced to the initial state, i.e. 0 h degradation time in humid air. Inset presents the maximum peak shifts recorded after the full 42 h, relative to 0 h, for the different peaks. (C) Peak shifts Δ* corrected by the accompanying changes in the α- phase band gap (essentially, Δ – E_g_). Inset: Band-gap corrected peak shifts at 26 h relative to 0 h. (**D)** Individual peak areas vs time with stretched exponential fits; average decay constant $\frac{\tau }{\beta }\left(\Gamma \left(\frac{1}{\beta }\right)\right)$ decreases systematically with increasing peak index. (**E), (F)** Schematic picture of electronic states within a finite quantum well: both a decreasing barrier offset and an increasing reduced effective mass lower the energies of the confined state.

We note that one other possibility for the observed red-shifts in the QC peaks is that the bandgap of the 3C α-phase of FAPbI_3_ may be evolving during degradation. As degradation progresses and the 3C (α) and 2H (δ) phases coexist, small shifts in the α-phase absorption edge may arise from strain variations within the remaining α domains. To probe whether the observed peak shifts are caused by such band gap changes, we applied band gap corrections to the previously extracted Δ values (Figure 3C). For this purpose, the optical gaps were obtained using the inflection-point method, yielding E_g_^INF^ from the steepest slope of the absorption onset (Figure S11). As shown in Figure S12, the band gap remains effectively constant during early degradation, with only a modest blue shift appearing after ∼ 26 h. At later times, however, increasing structural disorder broadens the absorption edge, rendering the corresponding band gap estimates less reliable. For this reason, the analysis is deliberately restricted to the initial degradation window (until 26 h). We find that after such changes in band gap have been accounted for (Figure 3C) the key trends are slightly weakened but remains qualitatively the same. The highest energy transition (Peak 5) continues to exhibit the largest redshift upon degradation, followed by the lower-energy features. This persistence shows that the spectral evolution is not driven by strain or disorder-induced band-edge shifts but instead reflects an intrinsic modification of the confinement potential and a genuine evolution of the quantum level structure during degradation. In addition, the temporal evolution of the integrated peak areas shown in Figure 3D indicates that the amplitude of the highest-energy transition (Peak 5) falls the most rapidly, followed progressively by those of lower-index states. This hierarchy combined with the ordering of the peak shifts, signals an electronic potential that steadily weakens as degradation progresses.

As discussed above, the peaked features attributed to QC may arise from either thin α-phase embedded within electronic barriers formed of higher-order hexagonal polytype (nH, with *n* > 2), or a subset of such polytypes themselves. While we are unable to make a definitive distinction, we note that for a finite electronic quantum well, a reduction in barrier height will lower the quantized state energies while simultaneously diminishing the effective confinement. Such weakening of quantum confinement would therefore be expected to naturally produce both the coordinated redshifts and selective loss of the least strongly bound transitions we observe (see Figure 3E for a schematic example). Any modest strain-induced increase in effective mass (m*), could also in principle shift the levels to lower energy (Figure 3F). However, it is not expected to weaken the confinement; instead, it effectively deepens the well and therefore cannot account for the ordered disappearance of the higher-energy states. Overall, the combined evolution of energies and amplitudes thus points to a confinement landscape that collapses as the α-phase and the accompanying network of higher-order polytypes disintegrates progressively in high humidity conditions. While the present measurements establish an ensemble-level correlation between the loss of QC features and the disappearance of short-range structural motifs, resolving the exact local stacking arrangement responsible for confinement will require the development of challenging imaging approaches capable of identifying such features on an atomic scale.

In summary, by following the QC features during structural degradation, we turn the α-to-δ transformation into a mechanistic test: structural motifs that persist while the QC peaks remain are plausible contributors, whereas motifs that grow as the peaks vanish are unlikely to contribute to their origin. Our optical and structural analyses reveal that changes in the high-energy peak features in the absorption of FAPbI_3_ during degradation result from the gradual collapse of electronic confinement, either within the α-phase domains or the polytypes. Sustained electronic confinement requires structural continuity within the α-phase network and a distinct potential contrast relative to surrounding higher-gap regions, or, if polytypes host these electronic signatures, their continued stability. As degradation progresses, these conditions deteriorate: the α-phase framework fragments, the interfacial potential contrast weakens, and the polytype structure begins to break down. Absorption data shows a strong correlation between the presence of the α-phase and the confined-state features, while the magnitude of 2H δ-phase is anticorrelated and therefore ruled out as a cause of the QC features. Meanwhile, XRD measurements reveal a simultaneous decline in both α-phase reflections and the presence of a broad background arising from small domains of higher-order hexagonal polytypes (nH, *n* > 2). These findings reveal a direct correlation between the presence of α-phase, such higher-order polytypes, and the high-energy absorption peaks. The hierarchical fading of spectral peaks, with higher-energy transitions decaying faster and experiencing a systematic redshift, is characteristic of a well-barrier system, where the confinement potential flattens as the contrast between well and barrier diminishes. This coordinated evolution supports the conclusion that all transitions arise from a single, confinement landscape that collapses as conversion to the 2H δ-phase proceeds. Together, these observations reveal that either α-phase quantum wells bordered by polytypic regions, or possibly the higher-order polytypes themselves, are the key structural elements responsible for the electronic states giving rise to the peaked absorption features in FAPbI_3_. While the exact role of the polytypes remains uncertain, their progressive reorganization is clearly linked to the disappearance of quantum-confined features. As we show in our study, such higher-order polytypic regions leave only a weak, broad diffuse background in XRD patterns suggesting a nanoscopic short-range origin, such as thin or faulted fragments embedded within the α-framework. Yet their impact on the absorption spectrum is strongly pronounced, leading to peak features whose presence has been shown to have deleterious effects on the performance of photovoltaics cells based on FAPbI_3_,^21^ although may prove useful for quantum applications.^19,21^ Our study therefore uses degradation as a diagnostic structural perturbation to identify the origin of these effects and highlights directions for their enhancement or elimination. Thus, the α- to δ- transformation is revealed here as a mechanistic probe of quantum confinement in FAPbI_3_, enabling the structural origins of the QC features to be isolated through their correlated evolution with the optical response.

## Supplementary Material



## Notes

Sample preparation, experimental method details, comparative linear decomposition over extended energy range, estimation of optical band gaps, trends exhibited by XRD peak intensities over time, reference XRD patterns, absolute shift in the peak positions, simulation of quantized states in a 1D quantum well (PDF)
