# Supplementary material for: Tracking the Breakdown of Quantum Confinement during Structural Degradation of FAPbI3
Source: J Phys Chem Lett. 2026 May 27;17(23):6566–73. doi: 10.1021/acs.jpclett.6c01253 (PMC13267083; doi:10.1021/acs.jpclett.6c01253)
Supplement: Supplementary file 1 [file jz6c01253_si_001.pdf]

Supporting Information

Tracking the Breakdown of Quantum Confinement during Structural Degradation of $FAPbI_3$

Gurpreet Kaur,[1] Sarah Scripps,[1] Joshua R.S. Lilly,[1] Nakita K. Noel,[1] Michael B. Johnston,[1] and Laura M. Herz[1,]*

[1] Department of Physics, University of Oxford, Clarendon Laboratory, Oxford OX1 3PU, United Kingdom

AUTHOR INFORMATION

Corresponding Author

*laura.herz@physics.ox.ac.uk

Table of Contents

1. Sample Preparation 3

2. X-Ray Diffraction (XRD) measurements 3

3. Steady State Optical Absorption Measurements 4

Deducing the absorption coefficient (α) from the measured reflectance (R) and transmittance (T) 5

Linear Decomposition 6

4. Comparison of the background signal for bare quartz substrate and that from the $FAPbI_3$ film 12

5. Trends exhibited by the different peak intensities in XRD over time 13

6. Reference XRD patterns 15

7. Shift in the peak positions – absolute values 16

8. Inflection point method to extract absorption onset energy 16

8. Simulation to show the effect of degradation on the quantised states inside a 1D quantum well 19

1. Sample Preparation

Materials:

Lead Iodide (PbI_2, 99.99%) was purchased from Tokyo Chemical Industry Ltd and Formamidinium Iodide (FAI, 99.99%) was purchased from Greatcell Solar. All materials were used without further purification.

Thin Film Fabrication:

Precursor ink for $FAPbI_3$ was prepared using a stoichiometric mixture of precursor powders in a 1:4 volume ratio of N - Methylpyrrolidone (NMP) to dimethylformamide (DMF) and left to stir in a glovebox overnight at room temperature.

Quartz substrates were cleaned by sequential sonication in detergent (Decon90 at 1% volume in deionised water), deionised water, acetone and isopropanol for 5 minutes each. After drying with N_2 gas, the substrates underwent UV-ozone treatment for 10 minutes.

Thin films were fabricated by statically depositing 50 μL of the precursor ink onto quartz substrates, followed by spin-coating at 6000 rpm. After 5 s of spinning, 300 μL of diethyl ether (DE) was rapidly dispensed to induce crystallization, and the resulting films were annealed at 150 °C for 15 min. All fabrication steps were carried out inside a nitrogen-filled glovebox to prevent moisture and oxygen exposure.

2. X-Ray Diffraction (XRD) measurements

Diffraction measurements were conducted using two complementary X-ray diffractometers to comprehensively assess the structural evolution of the $FAPbI_3$ films. The complete degradation series was recorded using a PANalytical X'Pert PRO powder diffractometer (Cu $K\alpha_1$, λ = 1.5406 Å) operated at 40 kV and 40 mA in conventional θ - 2θ geometry with continuous sample spinning, providing orientation-averaged diffraction patterns with stable intensity

scaling. Prior to analysis, the diffraction data were background-corrected using a third-order polynomial fitting procedure to minimize baseline contributions, and instrumental alignment was calibrated against the z-cut quartz reference peak at $2\theta = 16.43°$.

Complementary measurements were performed on a Rigaku SmartLab diffractometer equipped with a Cu source and a HyPix-3000 two-dimensional detector. In this fixed-detector configuration, set to capture the first-order reflections of both the α- and δ-phases, the large detector area provides broad angular acceptance, enabling the detection of weak off-axis scattering and making diffuse signals from stacking irregularities and polytypic disorder readily observable. To maximise the intensity of such weak signals, the SmartLab diffractometer was operated without a monochromator. Because the sample was not spun and had to be remounted for each acquisition, orientation dependent variations limit quantitative reproducibility, making this setup best suited for qualitative assessment of local structural disorder in the present study. In contrast, the PANalytical instrument, operated in Bragg–Brentano geometry with narrow receiving optics and continuous sample spinning, averages over orientation effects and provides quantitatively reliable diffraction profiles, while still capturing a reduced fraction of the diffuse intensity. Together, the two configurations offer complementary insight into the evolving structural hierarchy during degradation.

3. Steady State Optical Absorption Measurements

A Fourier Transform Infrared (FTIR) Spectrometer from Bruker (Model – Vertex 80v) was used to acquire the Transmittance (T) and Reflectance (R) from the films. The NIR lamp was used as the incident light source in combination with a Silicon diode as the detector. All the measurements were done under vacuum conditions (4mbar).

Deducing the absorption coefficient (α) from the measured reflectance (R) and transmittance (T)

From measurements of the photon-energy dependence of T and R plots, we extracted the absorption coefficient spectra (α) for the films utilising the following relationship:[1]

$$\alpha = \frac{-1}{d} \ln\left(\frac{T}{1-R}\right) \qquad \text{Eq. S1}$$

In this expression, d denotes the thickness of the $FAPbI_3$ layer deposited on the quartz substrate, T is simply T_{sample}/ T_{quartz} and similarly R is R_{sample}/ R_{mirror}. A silver mirror is used as reference for reflectance. The film thickness for all samples was measured to be approximately 150 nm, as determined using a Veeco Dektak 150 surface profilometer.

Linear Decomposition

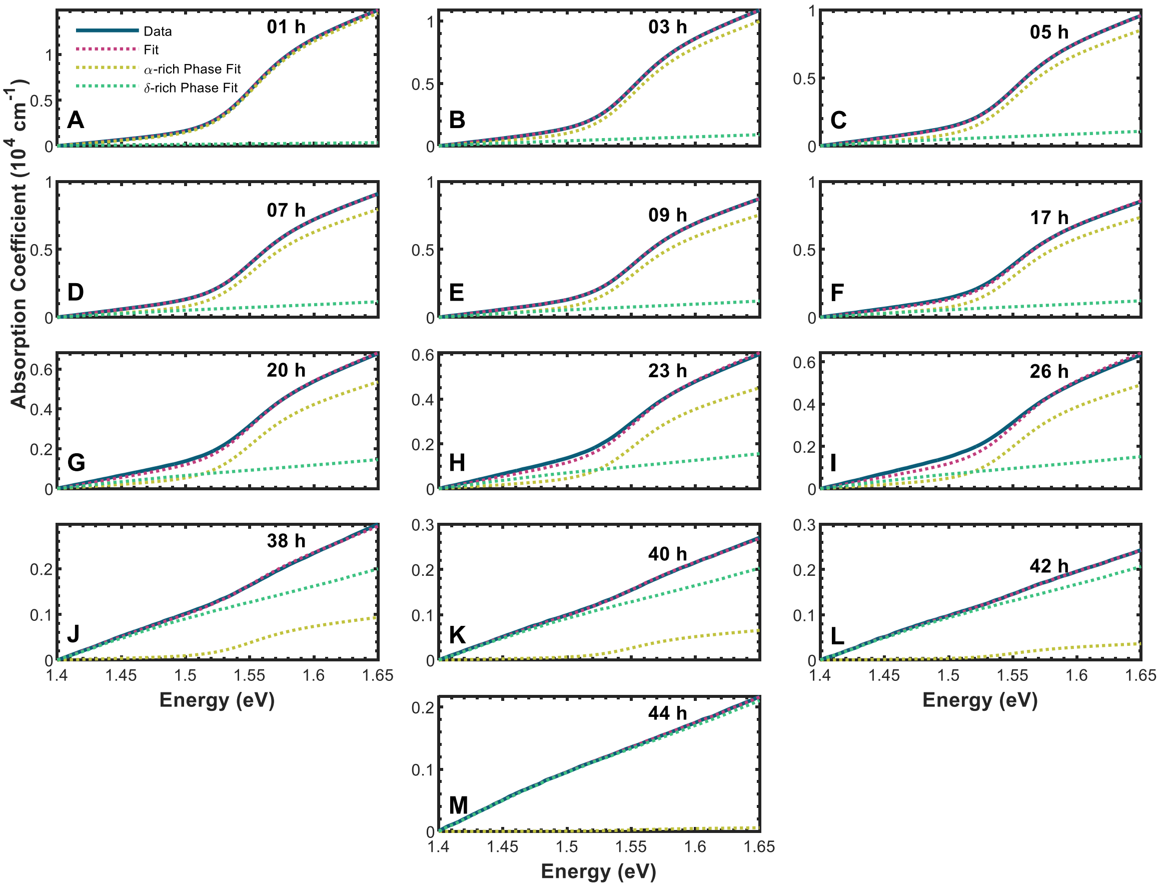

Figure S1. Linear decomposition of absorption coefficient spectra of a $FAPbI_3$ film during degradation. Each panel corresponds to a specific aging time and shows the absorption spectrum obtained from the experimental data expressed as absorption coefficient (navy blue) together with the reconstructed fit (magenta) obtained by linear combination of initial α-phase rich and the final δ-phase rich degraded endmembers (shown in Figure S2). The corresponding α-rich and δ-rich optical contributions are shown as yellow and green dotted curves, respectively.To quantify the relative phase contributions during the transformation process, each time-resolved absorption spectrum was expressed as a weighted linear combination of two reference spectra representing the α-phase (initial state, 0 h) and the degraded 2H δ-phase rich state (final state, 47 h). The decomposition was performed within the photon energy range

of 1.4 – 1.65 eV for reasons mentioned in the main text. The fitting procedure was constrained such that the sum of the phase coefficients equalled unity, ensuring a physically consistent representation of the mixed-phase system.

Each spectrum for the intermediate degradation stage is treated to be a linear combination of two known reference spectra:

$$\text{Fit} = X\cdot(\alpha\text{- rich Phase Fit}) + Y\cdot(\delta\text{- rich Phase Fit}) \text{ with the constraint: } X + Y = 1$$

X, Y imply the fraction of each phase.

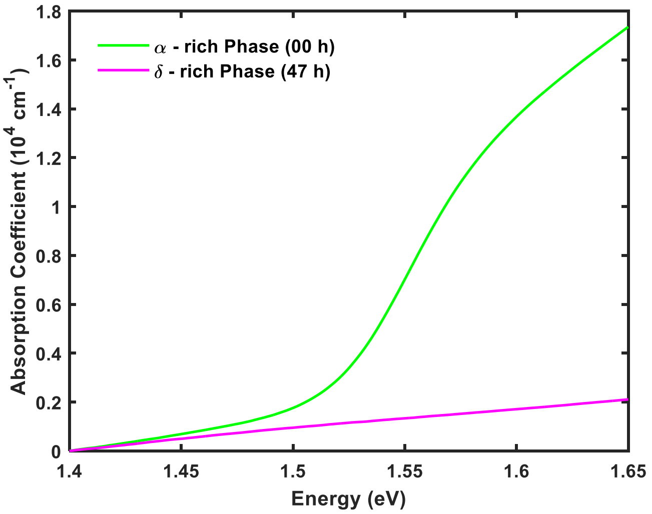

Figure S2. Absorption coefficient spectra of the reference endmembers used for the linear decomposition in Figure S1.

Supporting Table S1. Temporal evolution of the relative α-rich and δ-rich optical contributions in a $FAPbI_3$ film, extracted from linear decomposition of the absorption coefficient spectra using fixed

optical endmembers for the initial α-rich state and final δ-rich degraded state within the 1.4 -1.65 eV range.

Time (h)	α- rich phase	δ- rich phase
00	1.000	0.000
01	0.836	0.164
03	0.576	0.424
05	0.491	0.509
07	0.458	0.542
09	0.434	0.566
17	0.425	0.575
20	0.308	0.692
23	0.259	0.741
26	0.283	0.717
38	0.054	0.946
40	0.038	0.962
42	0.021	0.979
44	0.003	0.997
47	0.000	1.000

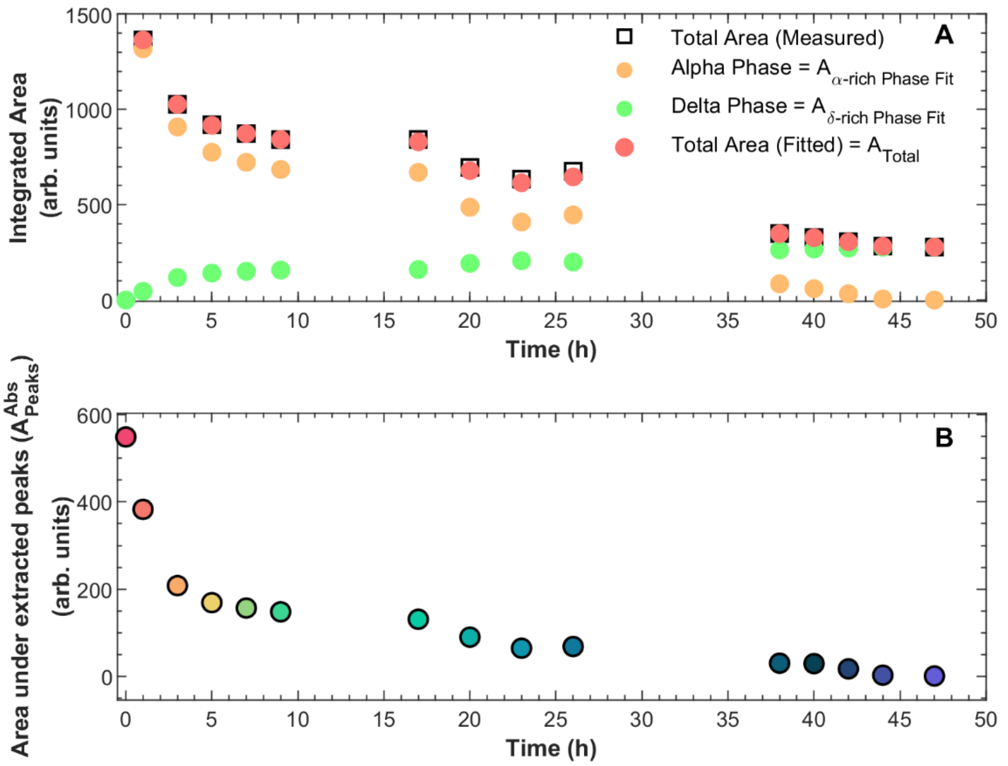

Figure S3. (A) Integrated absorption areas obtained from the linear decomposition of $FAPbI_3$ absorption spectra during aging, showing the α-rich optical contribution, δ-rich degraded-state optical contribution, and their reconstructed total contribution. The close agreement between the measured and fitted areas confirms the robustness of the decomposition method. The energy window for the area calculation here is confined near the absorption edge (1.4 – 1.65 eV) to focus on near band-edge variations. **(B)** Temporal evolution of the integrated area under the extracted quantum confinement features (A_{Peaks}^{Abs}). The energy window for this calculation has been selected to encompass the first and last minima of the extracted features in Figure 1B.

To further assess the robustness of this optical decomposition, the same constrained linear-combination procedure was repeated over an extended photon-energy range of 1.4–3.0 eV. This range includes the higher-energy absorption region where the δ-rich degraded film state contributes more strongly.

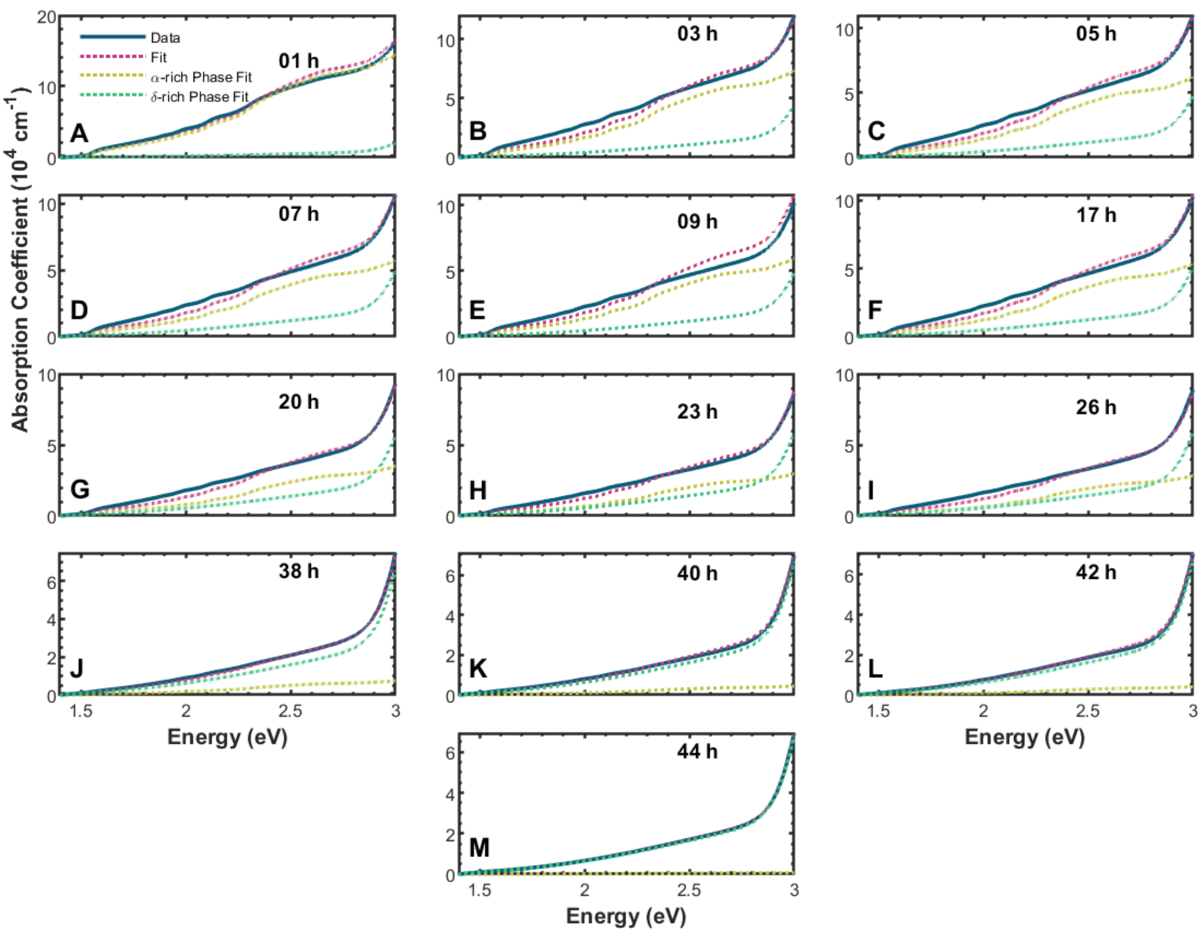

Figure S4. Alternative optical decomposition approach taken over an extended spectra range. The linear-combination analysis was repeated over a broader spectral window, 1.4-3.0 eV, to include the higher-energy region where the δ-rich degraded state contributes more strongly. The experimental absorption coefficient spectra are shown in navy blue, the reconstructed fits in magenta, and the corresponding α-rich and δ-rich optical contributions as yellow and green dotted curves, respectively. This analysis confirms that the qualitative temporal evolution of the optical endmember contributions is retained when the fitting range is expanded beyond the near-band-edge window used for the data and fits presented in Figure S1.

The same initial α-$FAPbI_3$-rich spectrum at 0 h and final δ-rich degraded-state spectrum at 47 h were used as fixed optical endmembers, and the coefficients were constrained to sum to unity. This extended-range (1.4-3.0 eV) analysis gave similar results (Figure S5) to those obtained

for the decomposition analysis across the spectral range near the band edge (1.4-1.65 eV, see Figure 1D in the main text).

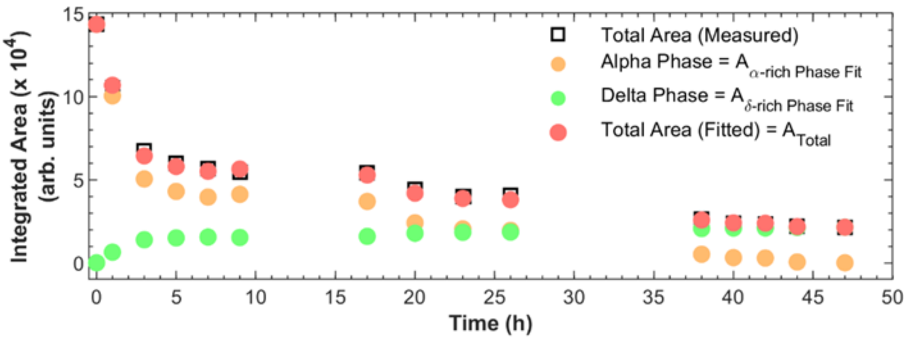

Figure S5. Extended-range area analysis and comparison with QC features. (A) Integrated spectral areas of the α-rich optical contribution, δ-rich degraded-state optical contribution, and reconstructed total absorption obtained from the extended-range linear decomposition. This analysis follows the same procedure as in the main text but uses the extended 1.4–3.0 eV fitting/integration range to assess whether the relationship between the QC features and the evolving optical endmember contributions is retained beyond the near-band-edge window.

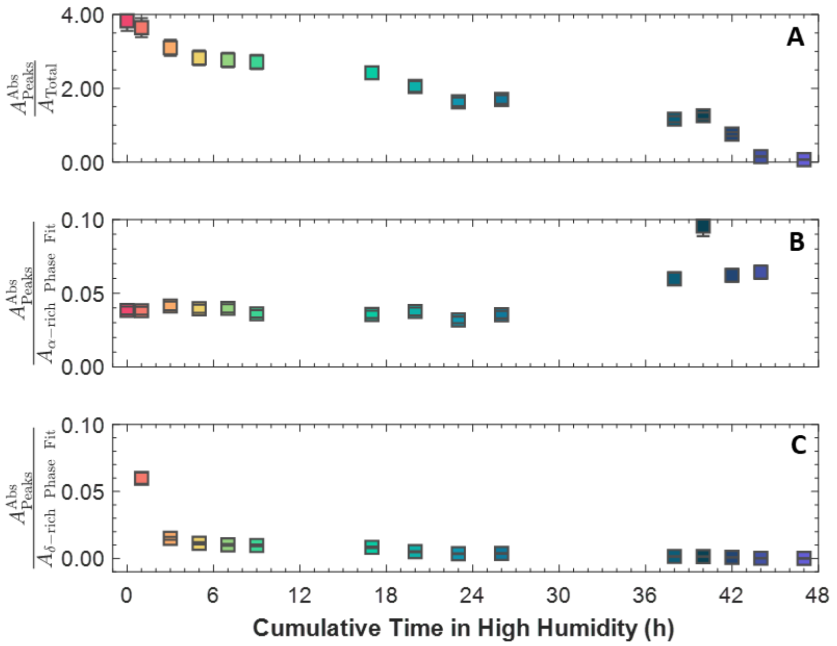

Figure S6: Time evolution of the spectrally integrated QC peak contribution normalized by different optical reference quantities during the degradation of $FAPbI_3$. This analysis follows the same procedure used in the main text for the restricted 1.4-1.65 eV fitting window, but is here applied to the extended 1.4-3.0 eV spectral range. For clarity, the values in panel A are scaled by 10^{-3}, and those in panel B are scaled by 10^{-1}.

4. Comparison of the background signal for bare quartz substrate and that from the $FAPbI_3$ film

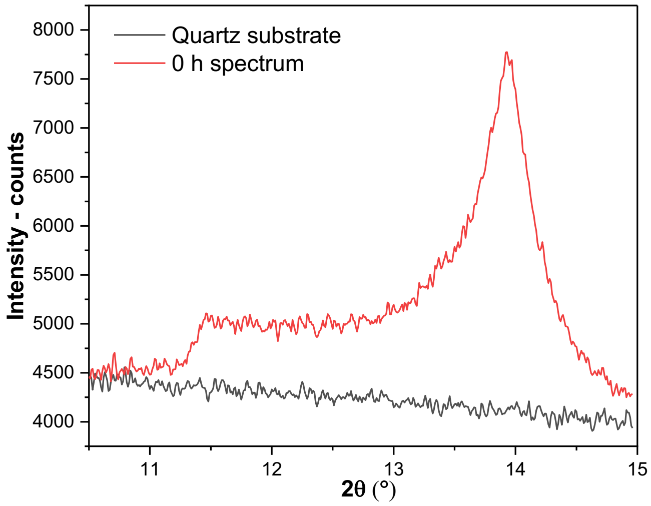

Figure S7: Comparison of the X-ray diffraction signal from a bare quartz substrate (black) and an as-prepared $FAPbI_3$ (0h) film (red), showing that the broad diffuse feature in the film is not present in the substrate/background measurement.

5. Trends exhibited by the different peak intensities in XRD over time

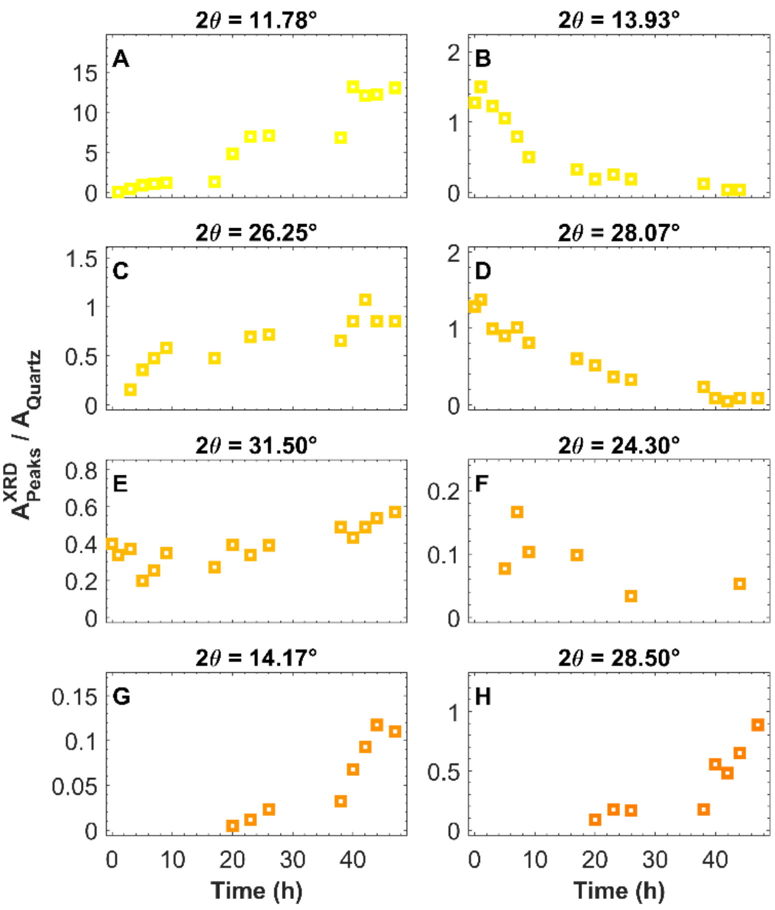

Figure S8. Time-dependent evolution of normalized XRD peak intensities for selected reflections in a $FAPbI_3$ film.

To monitor the structural evolution of the films during degradation, the integrated intensities of the characteristic XRD peaks corresponding to different reflections were analysed as a function of time (Figure S8 A–H). For each reflection, the peak area (A_{Peaks}^{XRD}) was normalized to the intensity of the quartz substrate reference peak (A_{Quartz}) to account for any instrumental or alignment variations during measurements. This normalization allows a direct comparison

of relative phase transformation kinetics across the monitored reflections. In a few cases, peak fitting failed, which limited the number of points used for calculating the peak area.

The resulting temporal profiles reveal distinct trends among different diffraction peaks. The reference patterns are provided in Figure S9. Reflections in panels A and C exhibit a progressive increase in relative intensity, indicative of the growth of the emerging 2H δ phase during the degradation process. For panel E, the observed trend reflects a combined contribution from the degrading perovskite α-phase and the concurrently growing δ-phase fraction, as the reflection centred at 31.5° can be attributed to both phases. In contrast, other reflections (panels B, D, F in Figure S8) show a continuous decline, consistent with the gradual depletion of the parent α phase. This complementary behaviour collectively confirms a phase transformation pathway involving the concurrent reduction of the initial α-phase and the formation of δ-phase under the applied environmental conditions with small traces of hydrates appearing only at later stages (panels G and H).

6. Reference XRD patterns

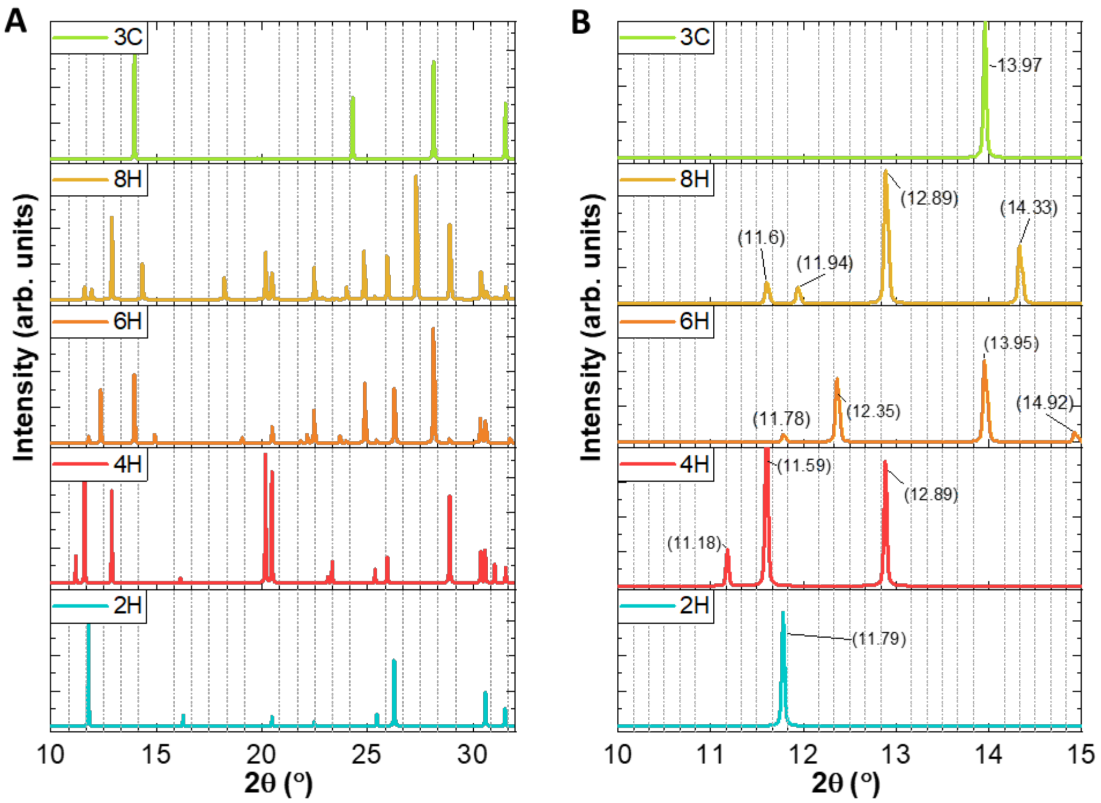

Figure S9. Reference patterns simulated using the cif files from literature[2] (with the incident X-ray wavelength set to Cu- kα_1).

For comparison, reference diffraction patterns corresponding to the α-phase (3C), δ-phase (2H), and several experimentally reported higher-order polytypic phases (nH, where n = 4, 6, and 8) were generated from crystallographic information files available in the literature and visualized using the VESTA software.[2]

7. Shift in the peak positions – absolute values

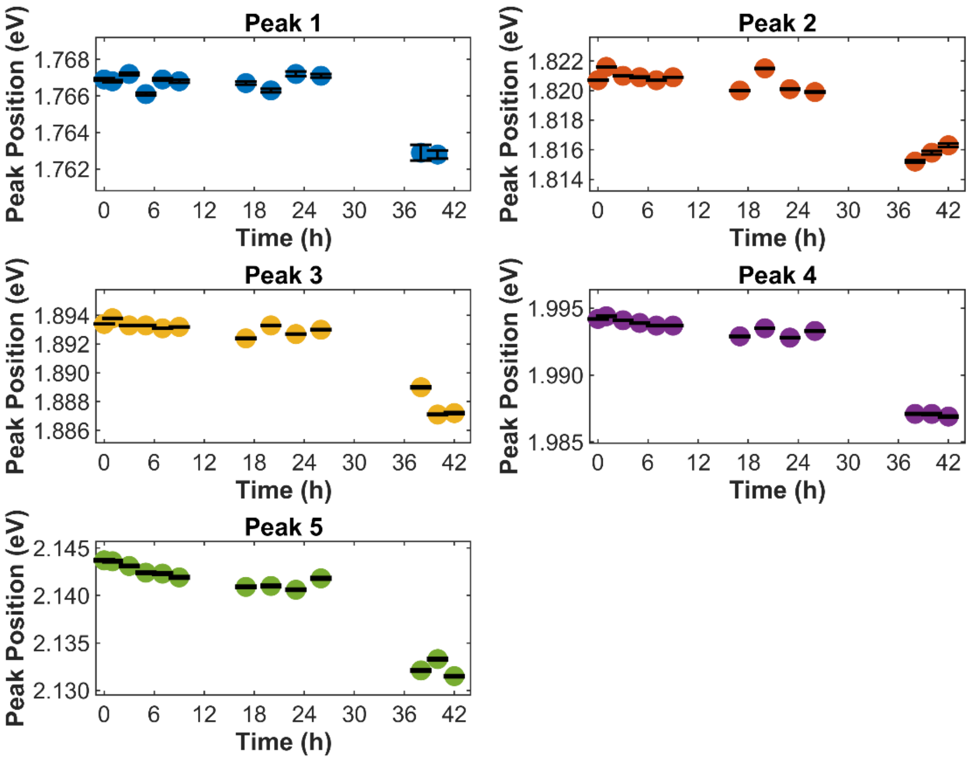

Figure S10. Absolute shifts in the energetic positions of peak features extracted from absorption measurements of a $FAPbI_3$ film, with black error bars indicating the associated uncertainties.

To stabilise the endpoint estimates, each point was averaged with its nearest neighbouring time point before evaluating the net shift described in the main text. Data beyond 42 h were omitted because the amplitude of the lowest index transition became too small to determine its position reliably, preventing consistent tracking of all peak shifts.

8. Inflection point method to extract absorption onset energy

The optical band gap (E_g^{INF}) at each stage of degradation was determined using the point of inflection method, which locates the band edge from the maximum slope of the absorption onset. The absorption coefficient α(E) was fitted with a smooth polynomial function, and the

optical band gap was taken as the energy at which the first derivative, dα/dE, reaches its maximum. Uncertainties were estimated by Monte Carlo resampling of the experimental data.

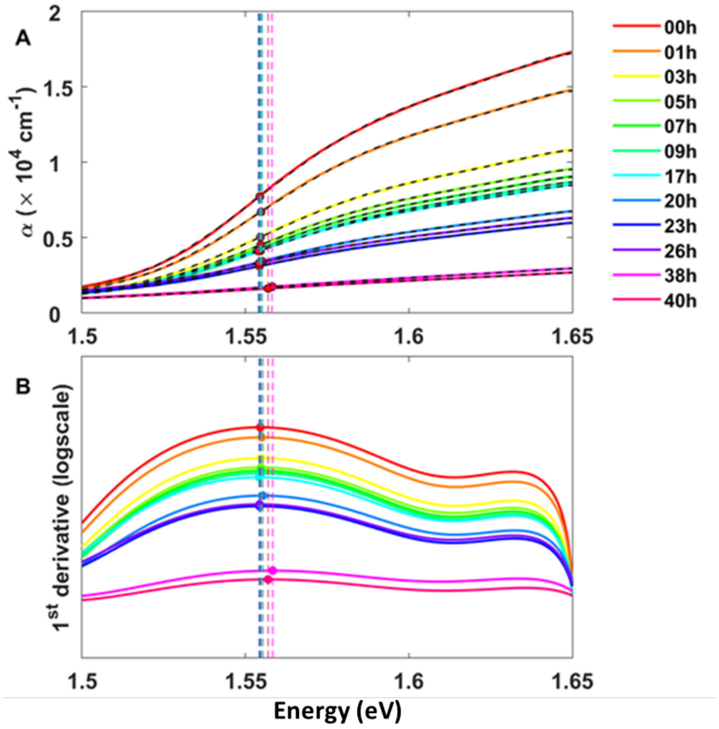

Figure S11. (A) Absorption coefficient spectra of $FAPbI_3$ films recorded during exposure to 85% relative humidity, with vertical dashed lines indicating the inflection points used to determine the optical band gap via the inflection point method. **(B)** First derivative spectra of the fitted absorption curves, where the peak maxima correspond to the energies of the absorption onsets used to determine the band gap.

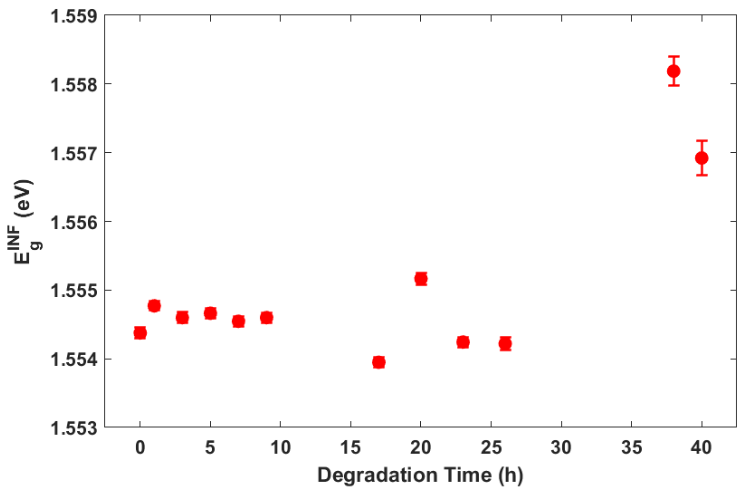

Figure S12. Optical band gap values (E_g^{INF}) for a $FAPbI_3$ film, extracted using the inflection-point method from the absorption spectra shown in Figure S11.

8. Simulation to show the effect of degradation on the quantised states inside a 1D quantum well

Theoretical Model and Energy Level Calculation

The infinite potential well is a useful conceptual illustration of confinement, as it highlights the characteristic $E_n \propto n^2$ scaling of the quantized levels with the quantum number n, a trend that is also reflected in the experimental energy spacing of the confined states observed here. However, an infinite well is not physically realistic, since actual semiconductor structures possess finite barriers that allow partial wavefunction penetration into the surrounding regions. A finite potential well model is therefore required to capture the relevant confinement physics.

To examine the quantized states of the proposed quantum well and the influence of barrier height and effective mass, charge-carrier confinement was modelled using a one-dimensional finite square well. This approach is used solely as a simplified framework for physical insight, as the parameter values employed are necessarily approximate and arbitrary. In this framework, low-energy states form discrete, well-confined levels, while higher-energy states increasingly penetrate the barriers and approach the continuum, deviating from idealized n^2 scaling and becoming more sensitive to changes in the potential. The analysis is based on the time independent Schrödinger equation, which describes the stationary states of an electron in a potential landscape $V(z)$

$$-\frac{\hbar^2}{2m^*}\left(\frac{d^2\psi(z)}{dz^2}\right) + V(z)\psi(z) = E\psi(z) \qquad \text{Eq. S2}$$

where $\hbar$ is the reduced Planck constant, m^* denotes the electron effective mass, $\psi(z)$ is the wavefunction, and E represents the energy eigenvalue associated with the bound state.

The potential profile $V(z)$ describes a finite square well, given by[3]

$$V(z) = V_{upper}, |z| > \frac{L}{2} \qquad \text{Eq. S3}$$

$$= V_{lower}, |z| \leq \frac{L}{2}$$

where V_{lower} corresponds to the potential energy within the well ($V_{lower} = 0$) and V_{upper} defines the barrier height outside the well. This form represents a quantum well structure with a region of low potential energy (the well) of width 'L' surrounded by higher potential barriers, mimicking a structure as expected in the system being investigated here.

Numerical Solution and Energy Evaluation

Since no analytical solution exists for the finite potential well, the Schrödinger equation has been solved numerically. The spatial coordinate z is discretized into uniform steps of size Δz, converting the continuous differential equation into a matrix eigenvalue problem. The second derivative in the kinetic energy term (the first one) is approximated using the central finite-difference method:

$$\frac{d^2\Psi(z)}{dz^2} = \frac{\Psi_{i+1} - 2\Psi_i + \Psi_{i-1}}{(\Delta z)^2} \qquad \text{Eq. S4}$$

Substituting this into the Schrödinger equation yields a discrete Hamiltonian operator H, represented by a tridiagonal matrix:

$$\mathrm{H}_{ij} = \begin{cases} \frac{\hbar^2}{m^*(\Delta z)^2} + V_i, & i = j \\ -\frac{\hbar^2}{m^*(\Delta z)^2}, & |i-j| = 1 \\ 0, & otherwise \end{cases} \qquad \text{Eq. S5}$$

where $V_i = V(z_i)$ is the potential energy at each spatial grid point z_i.

The energy eigenvalues E_n are then obtained by solving the matrix equation:

$$H\psi_n = E_n\psi_n \qquad \text{Eq. S6}$$

The energy eigenvalues E_n correspond to the quantized energy levels of the electron within the potential well, while the associated eigenvectors ψ_n represent the spatial wavefunctions of each bound state. Only the eigenvalues satisfying $V_{\mathrm{lower}} < E_n < V_{\mathrm{upper}}$ are physically meaningful, as they correspond to bound states confined within the well. The computed energies are reported in electron-volts (eV) following conversion from Joules for ease of physical interpretation.

Simulation Parameters

The numerical grid spanned a spatial range of 24 nm with a step size of 0.1 nm. The well width was set to L = 10 nm, chosen to approximate the expected confinement length scale in the QC domains.[4] The barrier heights were selected and varied arbitrarily for illustrative purposes, and the resulting trends should therefore be interpreted qualitatively. Two sets of parameter variations were considered:

1. **Barrier height dependence:**

The height of the potential barrier is varied between 0.15 eV and 0.25 eV, while the well width is fixed at 10 nm and the effective mass is taken as 0.1 m_e[4] (known reduced effective mass for $FAPbI_3$) to reflect the case of charge carriers being hosted inside thin layers of α-$FAPbI_3$. This example illustrates how altering the confinement barrier modifies the energy of the quantized levels.

2. **Variations in Effective Mass:**

For a fixed well width of 10 nm and a constant barrier height of 0.25 eV, the effective mass is varied between 0.1 m_e and 0.14 m_e. While 0.1 m_e corresponds to the value commonly reported for electrons in α-$FAPbI_3$,[4] the broader range is introduced solely for illustrative purposes, to assess the sensitivity of the quantized energy levels to modest changes in carrier mass. This calculation demonstrates how an increase in effective masses leads to a red shift in the bound states. The variation is deliberately modest, included to emulate any strain-induced changes in the effective mass, should such effects be present.

References

(1) Du, J.; Righetto, M.; Kober-Czerny, M.; Yan, S.; Elmestekawy, K. A.; Snaith, H. J.; Johnston, M. B.; Herz, L. M. Inter-Layer Diffusion of Excitations in 2D Perovskites Revealed by Photoluminescence Reabsorption. *Adv. Funct. Mater.* **2025**, *35* (26), 2421817. https://doi.org/https://doi.org/10.1002/adfm.202421817.

(2) Marchenko, E. I.; Fateev, S. A.; Korolev, V. V; Buchinskiy, V.; Eremin, N. N.; Goodilin, E. A.; Tarasov, A. B. Structure-Related Bandgap of Hybrid Lead Halide Perovskites and Close-Packed $APbX_3$ Family of Phases. *J. Mater. Chem. C Mater.* **2022**, *10* (44), 16838–16846. https://doi.org/10.1039/D2TC03202C.

(3) Hall, B. C. A Particle in a Square Well. In *Quantum Theory for Mathematicians*; Hall, B. C., Ed.; Springer New York: New York, NY, 2013;109–122. https://doi.org/10.1007/978-1-4614-7116-5_5.

(4) Wright, A. D.; Volonakis, G.; Borchert, J.; Davies, C. L.; Giustino, F.; Johnston, M. B.; Herz, L. M. Intrinsic Quantum Confinement in Formamidinium Lead Triiodide Perovskite. *Nat. Mater.* **2020**, *19* (11), 1201–1206. https://doi.org/10.1038/s41563-020-0774-9.
